# AI for Psychometrics: Validating Machine Learning Models in Measuring Emotional Intelligence with Eye-Tracking Techniques

**DOI:** 10.3390/jintelligence11090170

**Published:** 2023-08-22

**Authors:** Wei Wang, Liat Kofler, Chapman Lindgren, Max Lobel, Amanda Murphy, Qiwen Tong, Kemar Pickering

**Affiliations:** 1 The Graduate Center, City University of New York, New York, NY 10016, USA; 2 Brooklyn College, City University of New York, Brooklyn, NY 11210, USA; 3 Baruch College, City University of New York, New York, NY 10010, USA

**Keywords:** psychometric AI, emotional intelligence, machine learning, eye tracking

## Abstract

AI, or artificial intelligence, is a technology of creating algorithms and computer systems that mimic human cognitive abilities to perform tasks. Many industries are undergoing revolutions due to the advances and applications of AI technology. The current study explored a burgeoning field—Psychometric AI, which integrates AI methodologies and psychological measurement to not only improve measurement accuracy, efficiency, and effectiveness but also help reduce human bias and increase objectivity in measurement. Specifically, by leveraging unobtrusive eye-tracking sensing techniques and performing 1470 runs with seven different machine-learning classifiers, the current study systematically examined the efficacy of various (ML) models in measuring different facets and measures of the emotional intelligence (EI) construct. Our results revealed an average accuracy ranging from 50–90%, largely depending on the percentile to dichotomize the EI scores. More importantly, our study found that AI algorithms were powerful enough to achieve high accuracy with as little as 5 or 2 s of eye-tracking data. The research also explored the effects of EI facets/measures on ML measurement accuracy and identified many eye-tracking features most predictive of EI scores. Both theoretical and practical implications are discussed.

## 1. Introduction

AI—artificial intelligence—is a technology of creating algorithms and computer systems that can mimic human cognitive abilities to perform tasks (McCarthy 2007). With accelerating technological advances, the applications of AI techniques have been exploding in many industries, including but not limited to healthcare, finance, transportation, and entertainment. For example, AI has been used in medical services to detect neurodevelopmental disorders (Singh and Kaur 2020) and diagnose mental disorders (Yan et al. 2022). Some AI technologies have even become household products that serve our daily life (e.g., Siri in Apple smartphones, robot vacuums, etc.).

Unsurprisingly, AI has also changed psychometrics—the scientific discipline that studies the systematic measurement of psychological properties (Allen and Yen 2001). When AI techniques and methodologies were integrated with psychometric assessment testing, a new field was born: *Psychometric AI* (Bringsjord 2011; Bringsjord and Schimanski 2003). Indeed, AI algorithms can aid almost every aspect of psychometrics, from test development to test administration, from scoring to data analysis to building predictive models, etc.

Pioneering research in this field has demonstrated that integrating AI with psychometrics not only streamlines the assessment process and improves measurement accuracy, efficiency, and effectiveness (Chen et al. 2010; Hussein et al. 2019; Mujtaba and Mahapatra 2020) but also helps reduce human bias and increase objectivity in measurement (Altemeyer 2019; Wang et al. 2022). More importantly, early research that applied AI techniques in personality measurement has made significant breakthroughs in this new field. For example, Wu et al. (2015) found that machine learning (ML) models could more accurately judge personality traits than human beings (e.g., one’s friends). More recently, Berkovsky et al. (2019) leveraged eye-tracking techniques and AI-driven classifiers to predict self-reports of the HEXACO personality test scores and revealed that the measurement accuracy could be as high as 90%. 

Despite these exciting findings, researchers in this area seem to have overlooked an important individual difference characteristic—emotional intelligence, which is critical for every aspect of our social functioning. Mindful of this research gap, the current study aims to systematically examine the efficacy of various ML models in measuring different facets and measures of the emotional intelligence construct by leveraging fast-sampling eye-tracking big data and running about 15,000 ML models.

More specifically, the current study attempts to contribute to the literature on Psychometric AI for emotional intelligence by investigating four fundamental questions: (1) What is the level of accuracy that ML models can achieve in measuring emotional intelligence, and which ML model is the most effective in this endeavor? (2) Does ML accuracy differ across different facets or measures of emotional intelligence? Or can some facets/measures yield higher accuracy than others? (3) How much data do ML models require to achieve high accuracy in measuring emotional intelligence? In other words, if AI is powerful, can ML models accurately measure emotional intelligence with as little as 2 or 5 s of eye-tracking data instead of 10 s or more? (4) Lastly, if ML models can accurately measure emotional intelligence with eye-tracking data, what are the unique eye-tracking features most predictive of emotional intelligence measures?

We believe that answering these questions not only advances the scientific inquiry in the endeavor of Psychometric AI for emotional intelligence, but also sheds important light on the practical implications for the applications of emotional intelligence in management and education, given the significant role that emotional intelligence plays in both organizational and educational settings.

### 1.1. AI and Psychometrics

According to John McCarthy (2007), one of the founders of the discipline of artificial intelligence, AI is “the science and engineering of making intelligent machines, especially intelligent computer programs” (p. 2). It involves developing and creating computer systems and/or algorithm models that can perform tasks that would typically require human intelligence, such as learning from data, making decisions from the data, solving or predicting problems, and taking actions to achieve certain goals. The AI discipline encompasses several subfields, including machine learning, natural language processing, computer vision, and robotics.

Since the “AI Boom” in the 1980s that brought about tremendous government funding and research on the development of deep learning techniques (Tableau n.d.), AI technology has significantly developed and gained immense popularity in recent years. In addition, advancements in computing power, availability of large datasets, and breakthroughs in ML techniques have also fueled its growth. As a result, the applications of AI have exploded across numerous industries and functionalities, including, but not limited to, healthcare, finance, transportation, and entertainment. Indeed, hospitals and medical research have employed AI as clinical diagnostic and exploratory tools, which have allowed AI to recognize behavioral and cognitive traits that validly detect neurodevelopmental disorders (Singh and Kaur 2020). As AI can rely on visual, acoustic, verbal, and physiological features to train models that predict or aid in diagnosing mental disorders (Yan et al. 2022), AI can even compete with physicians by confirming or providing differential diagnoses. More importantly, due to the availability of large data storage and advancements in graphics processing units (GPUs) capable of processing millions of units of data with incredible efficiency, AI has essentially become part of household products in our daily life (e.g., Apple smartphones with built-in assistant Siri, robot vacuums such as Roomba, etc.).

### 1.2. Psychometric AI

Undoubtedly, AI is also revolutionizing the psychometric discipline. As a scientific discipline that studies the systematic measurement of psychological properties (Allen and Yen 2001), psychometrics can be AI-aided in almost every aspect, from test development to administration and from scoring to data analysis to building predictive models. Indeed, a new field—*Psychometric AI*, which integrates artificial intelligence techniques and methodologies with psychometric assessments and testing—is burgeoning. Bringsjord and Schimanski (2003) even argued that the purpose and definition of AI are essentially Psychometric AI, as the “I” part of “AI”, intelligence, is the primary focus of psychometrics and can be better understood from the standpoint of psychometrics. As such, Bringsjord (2011) defined Psychometric AI (or PAI) as:

“The field devoted to building information-processing entities capable of at least solid performance on all established, validated tests of intelligence and mental ability, a class of tests that includes not just the rather restrictive IQ tests…but also tests of artistic and literary creativity, mechanical ability, and so on”.

(p. 273)

Pioneer researchers in this area have explored the use of AI algorithms for automated scoring of tests (Chen et al. 2010; Hussein et al. 2019) and computerized adaptive testing (CAT; Mujtaba and Mahapatra 2020), natural language processing (NLP) for text analysis in measuring topical features (Wang et al. 2022), and more. These early studies have demonstrated that integrating AI with psychometrics not only streamlines the assessment process, but also improves measurement accuracy, efficiency, and effectiveness. For example, automated scoring systems have shown high reliability and agreement with human raters, and computerized adaptive testing has demonstrated improved efficiency and precision in estimating test-taker abilities (Hussein et al. 2019). More importantly, AI helps reduce human bias and increase objectivity in measurement (Altemeyer 2019).

In addition, researchers have begun directly employing AI techniques to measure personality traits and demonstrated promising results in this endeavor. For example, Wu et al. (2015) utilized LASSO (Least Absolute Shrinkage and Selection Operator) linear regression models to predict personality traits self-reported by over 70,000 participants on the 100-item International Personality Item Pool (IPIP) Five-Factor Model of personality questionnaire (Goldberg et al. 2006). They found that the ML models judged participants’ personality traits more accurately than the judgments made by the participants’ Facebook friends (Wu et al. 2015). More recently, Berkovsky et al. (2019) processed objective pupillometric data from an eye-tracking device and fed it into various AI-driven classifiers to predict self-reports of the HEXACO personality test (Thielmann et al. 2017). The accuracy was encouraging, ranging from 61.90 to 85.71% for image-only stimuli and 80.95 to 90.48% for image-video-combined stimuli. Similarly, Hoppe et al. (2018) applied ML methods to eye tracking during everyday behaviors (e.g., running an errand on a university campus) and found a considerable influence of personality on daily eye movement.

Despite the promising findings in Psychometric AI for personality traits, little research has been done to measure emotional intelligence in this area. Therefore, we conduct the current study to systematically examine the accuracy of AI models in measuring emotional intelligence.

### 1.3. Emotional Intelligence

Emotional intelligence (EI) is conceptualized as an ability to perceive and understand others’ and one’s own emotions and use this information to guide thoughts and behavior (Salovey and Mayer 1990). Analytical evidence from hierarchical and bifactor models also reveals that emotional intelligence is a second-stratum factor of intelligence (MacCann et al. 2014). Previous research has shown that EI is linked to a myriad of favorable outcomes, including physical and mental health (Zeidner et al. 2012), romantic relationship satisfaction (Malouff et al. 2014), and job satisfaction (Miao et al. 2017). There has also been a growing interest within Organizational Psychology in the application of emotional intelligence for its positive effect on job performance (Grobelny et al. 2021; Joseph et al. 2015) and leadership effectiveness amongst managers (Kerr et al. 2006).

Since its emergence in the early 1990s (Salovey and Mayer 1990), the construct of emotional intelligence was largely viewed as an ability similar to general or other cognitive abilities. Yet, the field of EI research has suffered from challenges relating to the theoretical validity and measurement of the construct. Later, researchers distinguished two EI constructs, Ability EI and Trait EI (Petrides and Furnham 2000). Ability EI is conceptualized as a set of abilities related to understanding emotions and is measured using performance-based tests with questions for which there are correct and incorrect answers, similar to IQ tests. On the other hand, Trait EI is conceptualized as a set of emotion-related dispositions, more akin to a personality trait, and is typically measured through self-report methods such as questionnaires (Petrides and Furnham 2000; Petrides et al. 2007a, 2007b). It has been suggested that Trait EI may be more indicative of typical behavior (Petrides and Furnham 2000), and thus it has been recommended that Trait EI measures be used in settings such as employment, where ongoing behavior is more likely to yield beneficial outcomes such as better job performance (Joseph et al. 2015; O’Connor et al. 2019).

Questions have been raised regarding the measurement accuracy of both methods. Tests of Ability EI rely on questions and problems that are deemed to have correct answers, which are typically determined by using general consensus and expert scoring methods. However, these two methods often yield contradictory results (e.g., Roberts et al. 2001). In addition, some researchers have questioned whether ability EI differs from general intelligence, and it has been shown that measures of ability EI are relatively weakly associated with outcomes they purport to be predictive of; in comparison, trait EI measures have been suggested to have better reliability and validity (see O’Connor et al. (2019) for a review). However, Trait EI measures, which overwhelmingly rely on individuals’ self-reports of their behavior and tendencies, are prone to biased or faking responses. For example, in high-stakes contexts such as job applications, individuals may fake their responses on self-reported measures in order to appear more desirable and qualified for a job (Birkeland et al. 2006).

As such, it is imperative to explore new methods to measure emotional intelligence that overcome the limitations and shortcomings of the traditional self-report approach. In addition, it is also important for the new measurement method to achieve similar or even higher accuracy. We believe that Psychometric AI—leveraging eye-tracking technology—may be an answer to the new measurement of emotional intelligence.

### 1.4. Eye-Tracking-Based Psychometric AI for EI Measurement

#### 1.4.1. The Eye-Tracking Technique

It has been suggested that the oculomotor system provides an indirect measure of brain activities and that eye-tracking measures can provide information about brain–behavior associations (Eckstein et al. 2017; Luna et al. 2008). Eye-tracking technology unobtrusively captures moment-by-moment ocular measures such as gaze fixation, eye movement, and pupil dilation while an individual attends to visual stimuli or engages with a task with a high degree of accuracy (Eckstein et al. 2017). With recent advances in technology, eye trackers have become relatively cost-effective, efficient, user-friendly, and easily portable, making them highly accessible and easy to use in many contexts (e.g., research labs, offices, hospitals, etc.). In fact, Cuve et al. (2022) demonstrated that a low-cost eye tracker was able to provide valid performance. Further, Wisiecka et al. (2022) found that the low-cost webcam-based eye tracker was viable. Consequently, eye tracking has increasingly been used in psychological research to study a variety of phenomena, including cognitive processes (Eckstein et al. 2017), psychopathology (Shishido et al. 2019), human interactions (Valtakari et al. 2021), and discrimination and stigmatization (Madera and Hebl 2012).

#### 1.4.2. Applications of Eye Tracking in Psychometrics, Affective Processing, and EI

Prior research has employed eye tracking to study individual differences, such as curiosity (Risko et al. 2012), aggressive tendencies (Laue et al. 2018), social anxiety (Konovalova et al. 2021), and self-esteem (Potthoff and Schienle 2021). Several studies have also found associations between the Big Five personality traits (i.e., agreeableness, conscientiousness, extraversion, neuroticism, and openness to experience) and eye-tracking measures (Al-Samarraie et al. 2018; Hoppe et al. 2018; Rauthmann et al. 2012). For example, Rauthmann et al. (2012) found specific patterns between eye-tracking parameters, such as dwell time and the number of fixations on specific stimuli, and extraversion, neuroticism, and openness, while agreeableness and conscientiousness were not related to eye-tracking measures. Because they observed relatively stable patterns among individuals’ gazing behaviors that differed from other individuals, they suggested that individual differences, specifically personality traits, may lead to specific gazing behaviors (Rauthmann et al. 2012).

On the other hand, psychologists have identified the connection between eye-tracking measures and affective processing. For example, eye-tracking research (Shasteen et al. 2014) studied and confirmed the “face in the crowd (FIC) effect”—threatening or angry target faces are identified more quickly and accurately than nonthreatening or happy target faces among a crowd of distractor faces. In addition, through both webcam-based and remote video eye tracking in three experimental conditions, Wisiecka et al. (2022) found that the time to first fixation toward happy faces was significantly shorter than toward sad faces in face-in-the-crowd (FIC) tasks. Similarly, in a study examining the mediating role of attention to positive faces in the relationship between Trait EI and affect, Suslow et al. (2022) found that emotion regulation was associated with fixation time on happy faces in a free-viewing paradigm where four emotional faces (angry, happy, sad, and neutral) were presented simultaneously.

More importantly, a few pioneering studies have also employed eye tracking methodology to study the association between EI and attention to emotional faces. For instance, Lea et al. (2018) found that trait EI was associated with visual preference for positive stimuli, as those with higher EI scores fixated longer on happy faces when those were presented in a crowd of mostly angry faces. Davis (2018) used a visual dot probe paradigm where emotional expressions (angry, happy, or sad) were paired with a neutral expression and showed a complex pattern of associations between EI (both Ability EI and Trait EI) and bias towards looking at emotional faces. For Trait EI, those higher in Sociability had a bias towards looking at angry faces, while Well-being was associated with avoidance of sad faces, but the latter was only found under a stress condition. For Ability EI, emotion management was associated with avoidance of angry faces (although this was only marginally significant). In contrast to other findings, there were no associations between EI and bias for happy faces. However, it is important to note that Davis (2018) used a different type of paradigm and assessed early attentional preference compared to sustained attention that was examined by Lea et al. (2018) and Suslow et al. (2022). 

#### 1.4.3. The Eye-Tracking-Based AI Model for EI and the Effect of Data Quantity on Model Performance

Nevertheless, there has been little research that combines eye-tracking techniques and Psychometric AI methods to investigate EI measurement. We believe that the combination of Psychometric AI methods and eye-tracking techniques provides an ideal approach to studying the psychometrics of EI. On the one hand, the aforementioned research has long noticed that EI is associated with various eye movements that can be precisely captured and quantified by eye-tracking measures. On the other hand, AI models can powerfully handle a large number of eye-tracking measures that would be otherwise challenging for traditional analytical methods. Thus, one of the goals of this study is to systematically examine the efficacy of various ML models in predicting different facets and measures of the emotional intelligence construct by using eye-tracking measures. 

Further, no prior research has explored the critical question of the effect of eye-tracking data quantity on AI model performance for EI measurement. That is, if AI is powerful, can ML models accurately measure emotional intelligence with a small amount of eye-tracking data? More specifically, can 5 or 2 s of eye-tracking data achieve measurement accuracy as high as 10 s of eye-tracking data? By answering this question, we believe we are able to novelly examine the power of AI models for EI measurement. 

Therefore, the current study aims to investigate the following set of four research questions:

RQ1: What is the level of accuracy that ML models can achieve in measuring emotional intelligence, and which ML model is the most effective in this endeavor?
RQ2: Does the ML accuracy differ across different facets or measures of emotional intelligence? Or can some facets/measures yield higher accuracy than others?
RQ3: How much data do ML models require to achieve high accuracy in measuring emotional intelligence?
RQ4: If ML models can accurately measure emotional intelligence with eye-tracking data, what are the unique eye-tracking features most predictive of emotional intelligence measures used in the ML models?

## 2. Method

### 2.1. Design and Participants

An experimental study was designed with two sessions: an eye-tracking session and a survey session. The two sessions were randomized to eliminate the potential order effect. The eye-tracking session involved two blocks of experimental tasks in which participants viewed visual stimuli with different emotional facial expressions. In the survey session, participants completed two self-report measures on emotional intelligence and demographic information. The visual stimuli and emotional intelligence measures are detailed in the next subsections.

The sample consisted of 218 adults (*M* = 26.79, *SD* = 11.73, 59.26% female) recruited from a university and the campus community in a large metropolitan area in the Northeast United States. The participants had diverse ethnic backgrounds (28.9% Asian/Pacific Islander, 23.9% Black, 19.3% Hispanic/Latino, 15.6% Caucasian/White, 5.0% multiracial, 3.2% other, and 4.1% declined/missing). The protocol was approved by the university Institutional Review Board. Participants received either partial course credit or monetary compensation in exchange for their participation.

### 2.2. Emotional Intelligence Measures 

We administrated two commonly used emotional intelligence measures in this study: the Wong and Law Emotional Intelligence Scale (WLEIS) and the short form of the Trait Emotional Intelligence Questionnaire (TEIQue-SF). 

#### 2.2.1. WLEIS

The WLEIS is a 16-item questionnaire scored on a 7-point Likert scale ranging from 1 (strongly disagree) to 7 (strongly agree; Wong and Law 2002). The questionnaire was designed for leadership and management research and consists of four subscales that measure the ability to appraise and express emotions (self-emotional appraisal or SEA; e.g., “I have good understanding of my own emotions”), the ability to appraise and recognize emotions in other people (others’ emotional appraisal or OEA; e.g., “I always know my friends’ emotions from their behavior”), the ability to use emotions for performance (use of emotion or UOE; e.g., “I always set goals for myself and then try my best to achieve them”), and the ability to regulate emotions (regulation of emotion subscale or ROE; e.g., “I have good control of my own emotions”). The reliability of the WLEIS was 0.88 in this study. Each subscale is comprised of four items which are averaged to generate a subscale score, and all 16 items in the questionnaire were aggregated to generate an overall WLEIS score.

#### 2.2.2. TEIQue-SF

The TEIQue-SF is a short-form version of the trait emotional intelligence measure developed by Petrides (2009). It is comprised of 30 items with four dimensions on a 7-point Likert scale. The four dimensions of the TEIQue-SF questionnaire include Well-being (6 items, e.g., “I feel that I have a number of good qualities”), Self-control (6 items, e.g., “On the whole, I’m able to deal with stress”), Emotionality (8 items, e.g., “Expressing my emotions with words is not a problem for me”), and Sociability (6 items, e.g., “I can deal effectively with people”). The reliability of TEIQue-SF was 0.89. Descriptive statistics for the two EI measures and the corresponding eight facets are presented in Table 1.

### 2.3. Visual Stimuli and Experimental Tasks

The experimental task was adapted from Lea et al. (2018) but used a different set of emotional facial stimuli—the NimStim Set of Facial Expressions, which uniquely used untrained individuals to rate the stimuli (Tottenham et al. 2009). The task consisted of two blocks in which visual stimuli of emotional faces were presented to participants.

**Figure 1:**
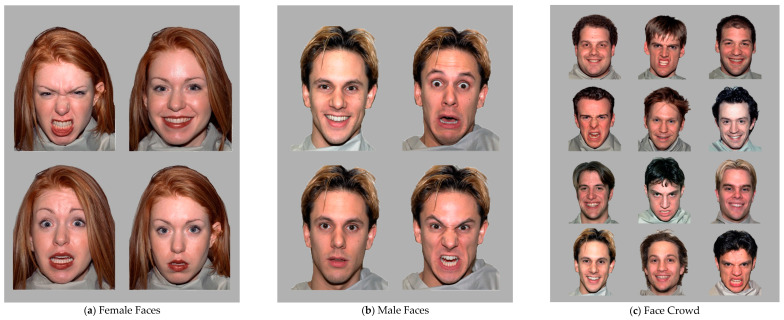
Sample Images of Facial Expression Stimuli. (**a**,**b**) show stimuli from the first block of the experimental task, whereas (**c**) shows a crowd stimulus from the second block.

The first block consisted of 20 trials, and in each trial, four faces of the same person portraying four different emotional expressions (angry, fearful, happy, and neutral) were presented simultaneously in a 2 × 2 matrix. The placement of each of the four expressions within the matrix (e.g., top left, top right, etc.) differed between trials. There were ten female-presenting and ten male-presenting faces of various ethnicities (see Figure 1a,b for examples). All faces were presented with a central direct gaze (Dalmaso et al. 2020).

The second block also consisted of 20 trials, which presented many emotional faces in a crowd with varying ratios of happy to angry faces. Specifically, in each trial, the crowd stimuli presented 12 faces of different people portraying either happy or angry expressions presented simultaneously in a 4 × 3 matrix (see Figure 1c for an example). The angry-to-happy ratios varied in five conditions: 2:10, 4:8, 6:6, 8:4, and 10:2. Each ratio was presented four times, with different placements of the expressions within the matrix at each trial. The crowd stimuli consisted of only Caucasian male-presenting faces, as prior research found that male faces were processed faster in this paradigm (Lange et al. 2011) and that people tend to look and explore their own race and other-race faces differently (Kawakami et al. 2022). 

**Figure 2:**
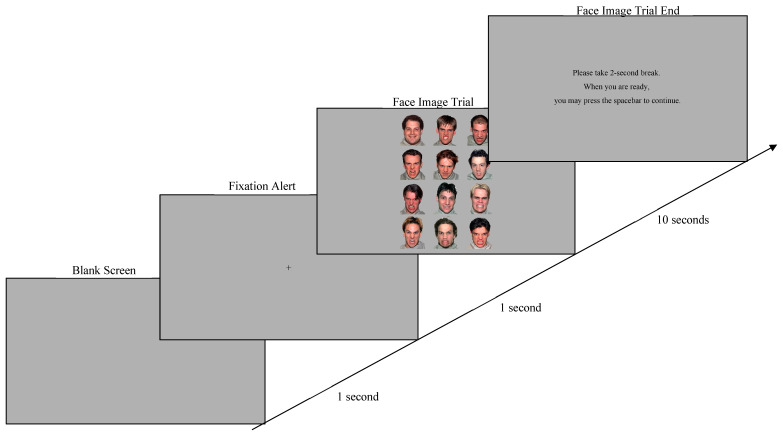
Experimental Sequence of Presenting Facial Expression Images.

The two blocks were presented in the same order for each participant, and the trials within each block were randomized across participants. For each trial, participants were first presented with a blank screen for 1 s, followed by a fixation screen with a cross (“+”) in the middle for another 1 s, which was then followed by facial stimuli for 10 s. Each trial ended with a screen instructing the participant to take a break for up to 2 s (see Figure 2). 

### 2.4. Eye-Tracker Device

Binocular recordings of eye movements were obtained at 1000 Hz using an EyeLink Portable Duo eye-tracking system (SR Research Ltd., Ontario, Canada) in head-stabilized mode. Calibration and validation were performed for each participant at the beginning of the eye-tracking task, and drift correction was performed throughout the task as needed. A total of 12 out of the 218 participants were excluded due to the inability to capture eye-tracking data caused by calibration and/or validation errors.

### 2.5. Analytic Strategy

The analysis consisted of three major steps. The first step was to generate eye-tracking measures from the eye-tracking data. This step required creating interest areas and specifying interest periods. We created an interest area for each face on each image with a freehand shape. Across the 40 images, there were 320 faces in total, so 320 interest areas were first created and labeled in 18 groups: female angry, female fearful, female happy, female neutral, male angry, male fearful, male happy, male neutral, 2-angry crowd, 4-angry crowd, 6-angry crowd, 8-angry crowd, 10-angry crowd, 2-happy crowd, 4-happy crowd, 6-happy crowd, 8-happy crowd, and 10-happy crowd. To determine if a small amount (i.e., a shorter duration) of eye-tracking data was sufficient to achieve similar accuracy to that of a large amount of eye-tracking data, we specified three interest periods: the entire 10 s, the first 5 s, and the first 2 s. After creating interest areas and periods, we then generated common eye-tracking measures (see a full list in Table 2). These eye-tracking measures were computed by using algorithms that were developed and validated by the eye-tracker manufacturer, SR Research Ltd. (2022). As one of the most popular eye-tracker brands for scientific research, SR Research eye trackers have been widely used in psychology and neuroscience and have generated more than 11,000 peer-reviewed publications (see https://www.sr-research.com/full-publications-list, accessed on 20 July 2023).

The eye-tracking measures, crossing 18 unique interest areas and 40 visual stimuli, generated over 200 eye-tracking predictors. We also included age and gender as predictors in the ML modeling. That is, each AI model received all the eye-tracking measures plus age and gender as the predicting variables. 

**Table 2:** Eye-Tracking Measures and the Descriptions by Different Focus Areas.

Measure	Description
Area in focus: The entire image in a trial	
Blink Count	The total number of blinks in the trial within an interest period
Average Saccade Amplitude	Average size (in degrees of visual angle) of all saccades in the trial within an interest period
Average Blink Duration	Average duration (in milliseconds) of all blinks in the trial within an interest period
Fixation Count	The total number of fixations in the trial within an interest period
Area in focus: Each interest area	
Average Fix Pupil Size	Average pupil size across all fixations in the interest area within an interest period
Dwell Time	The summation of the duration across all fixations on the current interest area within an interest period
Fixation Count	The total number of fixations falling in the interest area within an interest period
First Fixation Time	Start time of the first fixation to enter the current interest area within an interest period
FSA Count	The number of fixations on the current interest area from another certain interest area within an interest period in the fixation sequence analysis (FSA), e.g., how many times does a participant switch his/her fixation from the female angry interest area to the female happy interest area? Or vice versa?
Run Count	The number of times the interest area was entered and left (runs) within an interest period

The second step involved data management and combination. The eye-tracking measures generated in the first step were structured in a long format with about one million rows. For example, the fixation count measure was listed in 960 rows (i.e., 320 interest areas by 3 interest periods) for each participant. Thus, we needed to aggregate and reformat the data first, then combine the eye-tracking measures with the self-reported emotional intelligence data. Participants who failed to pass the attention checks (via five quality control items) were also excluded at this step.

After the data were processed, we proceeded with running seven classic machine learning models: Naïve Bayes, Support Vector Machine (SVM) linear models, non-linear SVM with a radial kernel function, non-linear SVM with a polynomial kernel function, k-Nearest Neighbors (KNN), Decision Tree (DT), and Random Forest (RF). In order to better examine the performance of machine learning models, we dichotomized the outcome variable (2 EI measure total scores and 8 facet subscores) at seven different percentiles: the 20th, 30th, 40th, 50th, 60th, 70th, and 80th. For each ML model and each outcome variable, we ran 10 times for cross-validation, where each time, 75% of the data was randomly selected for training and the remaining data were used for testing, and we then reported the average accuracy. We repeated the analysis for each of the three interest periods of eye-tracking data: 10 s, 5 s, and 2 s. Altogether, we performed 10 EI scores × 7 ML classifiers × 7 percentiles × 3 interest periods = 1470 ML runs.

## 3. Results

### 3.1. Descriptive Statistics of EI Measures

As presented in Table 1, the Wong and Law Emotional Intelligence Scale (WLEIS) and the Trait Emotional Intelligence Questionnaire (TEIQue-SF) were highly correlated (*r* = 0.70, *p* < .01). However, the facets of the WLEIS were only moderately correlated with facets of the TEIQue-SF, ranging from 0.22 (*p* < .01) to 0.66 (*p* < .01). The correlations between the TEIQue-SF total and score and the facets of WLEIS—SEA, OEA, UOE, and ROE—were 0.56, 0.39, 0.67, and 0.61, respectively; similarly, the correlations between the WLEIS total score and the facets of TEIQue-SF—Well-being, Self-control, Emotionality, and Sociability—were 0.59, 0.59, 0.52, and 0.43, respectively. These correlations were all statistically significant at the *α* = 0.01 level.

### 3.2. The Machine Learning Identification Accuracy

**Figure 3:**
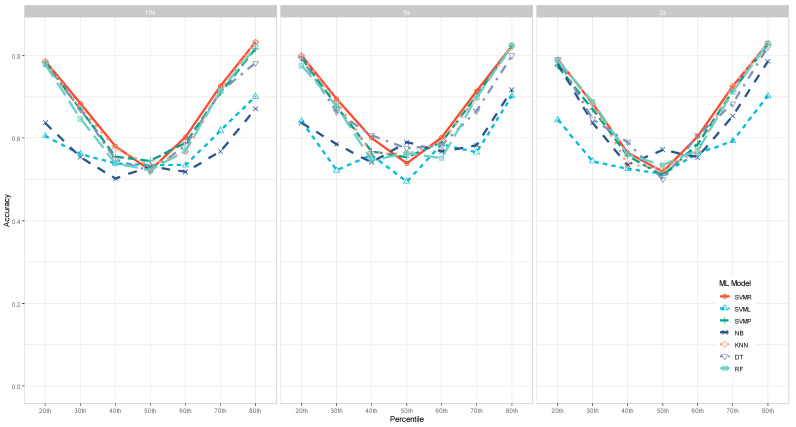
Performance of Machine Learning Models as a Function of Percentiles to Dichotomize Data. NB = Naïve Bayes models, SVM*_L_* = Support Vector Machine linear models, SVM*_R_* = Non-linear Support Vector Machine with a radial kernel function, SVM*_P_* = Non-linear Support Vector Machine with a polynomial kernel function, KNN = k-Nearest Neighbors models, DT = Decision Tree models; RF = Random Forest models.

Table 3 shows the accuracy results of 1470 ML runs we performed in this study. To better compare the performance of different machine learning models and identify the patterns, we aggregated the accuracy across the 10 EI scores and visualized it in Figure 3.

Across the three interest periods—10s, 5s, and 2s—a clear pattern emerged: The accuracy of ML models showed a “V” shape along the percentiles of how the EI outcome variable was dichotomized. When it was dichotomized at the median (i.e., the 50th percentile), the ML performance accuracy was the lowest—slightly above 0.50. However, the accuracy was the highest at the two ends when the data were dichotomized at either the 20th or 80th percentiles, indicating that ML models could accurately identify individuals with either the top or bottom 20% of EI scores. In addition, the accuracy of identifying the top 20% of EI scores was even higher than the accuracy of identifying the bottom 20% of EI scores. In general, the accuracy was between 50 and 60% when the dichotomy was in the middle range (e.g., the 40th–60th percentiles), and it boosted to about 0.70 to over 0.90 when the dichotomy was more extreme (e.g., 20th to 30th and 70th to 80th percentiles).

The performance of the seven ML models varied substantially. As shown in Figure 3, the Non-linear Support Vector Machine with a radial kernel function (SVM*_R_*) model was the most efficacious when the data were dichotomized at two ends (e.g., ≤40th or ≥60th). In contrast, the Naïve Bayes (NB) models and Support Vector Machine linear models (SVM*_L_*) generated the poorest results for such cases. However, the NB models performed relatively better compared to other ML models when the data were dichotomized at the 50th percentile.

### 3.3. The Effect of EI Facets/Measures on Machine Learning Identification Accuracy

**Figure 4:**
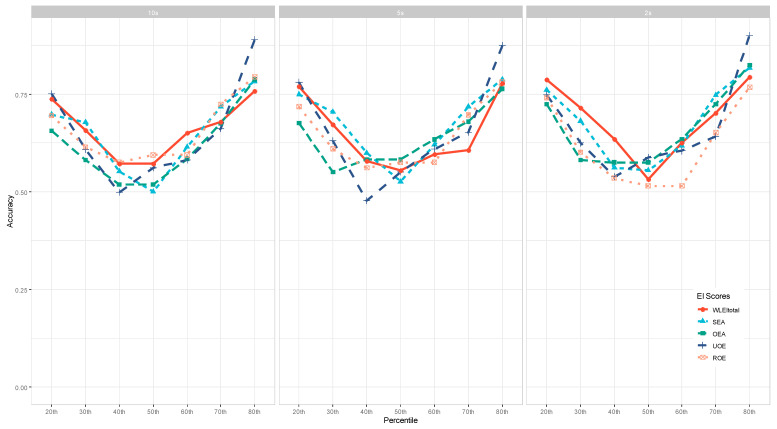
The Effect of WLEIS on Machine Learning Accuracy.

**Figure 5:**
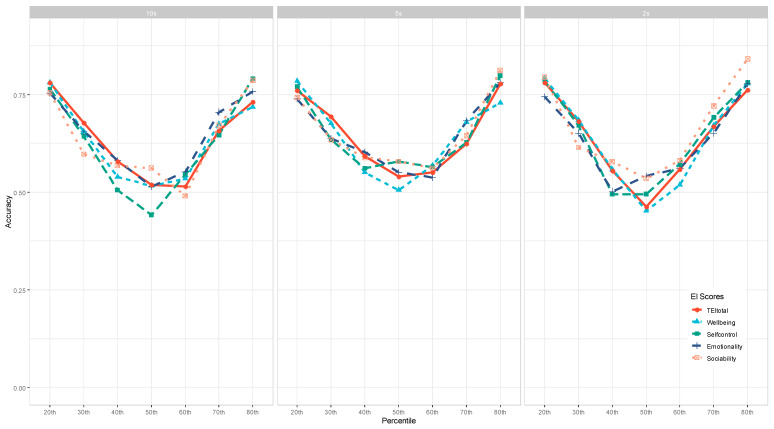
The Effect of the TEIQue-SF on Machine Learning Accuracy.

To examine the impact of EI facets and measures on ML identification Accuracy, we aggregated the accuracy across the 7 ML models and visualized the patterns for the WLEIS and TEIQue-SF measures separately. As shown in Figure 4 and Figure 5, there were no clear patterns in terms of which EI facet yielded better accuracy, depending on other conditions, such as interest periods and percentiles to dichotomize the data.

Across the three panels in Figure 4, the Use of Emotion (UOE) facet of the WLEIS achieved the highest accuracy (88–90%) when the data were dichotomized at the 80th percentile. When the dichotomy was cut off at the 40th percentile or lower, the WLEIS total scores achieved the highest accuracy for the 2 s interest period of eye-tracking data.

For the TEIQue-SF measure (see Figure 5), its total scores yielded the highest accuracy when the dichotomy cutoff was the 40th percentile or lower, whereas the Self-control facet seemed to achieve the lowest accuracy. When the dichotomy cutoff was the 60th percentile or higher, the Emotionality facet performed the best for the 10 s and 5 s interest periods, yet the Sociability facet performed the best for the 2 s interest period.

**Table 3:** Classification Accuracy for Different EI Facets/Measures by 7 Supervised ML Models with Different Amounts of Eye-Tracking Data.

	10 s									5 s									2 s							
	NB	SVM*_L_*	SVM*_R_*	SVM*_P_*	KNN	DT	RF	Mean		NB	SVM*_L_*	SVM*_R_*	SVM*_P_*	KNN	DT	RF	Mean		NB	SVM*_L_*	SVM*_R_*	SVM*_P_*	KNN	DT	RF	Mean
	20th Percentile																									
SEA	.605	.535	.767	.721	.767	.767	.721	**.698**		.683	.537	.805	.805	.805	.805	.805	**.749**		.767	.721	.767	.767	.767	.767	.767	**.761**
OEA	.636	.432	.705	.705	.705	.705	.705	**.656**		.585	.512	.732	.732	.732	.732	.707	**.676**		.721	.698	.721	.721	.721	.767	.721	**.724**
UOE	.674	.628	.791	.791	.791	.791	.791	**.751**		.659	.780	.805	.805	.805	.805	.805	**.780**		.744	.535	.791	.791	.791	.791	.791	**.748**
ROE	.500	.614	.750	.750	.750	.750	.750	**.695**		.585	.659	.756	.756	.756	.756	.756	**.718**		.721	.674	.767	.721	.767	.767	.767	**.741**
WLEIS Total	.581	.535	.814	.814	.814	.791	.814	**.738**		.634	.610	.829	.829	.829	.829	.829	**.770**		.837	.628	.814	.791	.814	.814	.814	**.787**
Well-being	.674	.721	.814	.814	.814	.814	.814	**.781**		.732	.610	.829	.829	.829	.829	.829	**.784**		.767	.721	.814	.814	.814	.814	.791	**.791**
Self-control	.674	.605	.814	.814	.814	.814	.814	**.764**		.634	.780	.805	.805	.805	.805	.756	**.770**		.814	.605	.814	.814	.814	.814	.814	**.784**
Emotionality	.659	.705	.795	.795	.773	.795	.750	**.753**		.634	.683	.805	.805	.805	.805	.634	**.739**		.791	.628	.791	.698	.791	.744	.767	**.744**
Sociability	.721	.535	.814	.814	.814	.767	.814	**.754**		.634	.585	.805	.756	.805	.805	.805	**.742**		.814	.651	.814	.814	.814	.814	.837	**.794**
TEI Total	.651	.744	.814	.814	.814	.814	.814	**.781**		.585	.659	.829	.829	.829	.756	.829	**.760**		.837	.581	.814	.814	.791	.814	.814	**.781**
**Sub-Mean**	**.638**	**.605**	**.788**	**.783**	**.786**	**.781**	**.779**	**.737**		**.637**	**.641**	**.800**	**.795**	**.800**	**.793**	**.776**	**.749**		**.781**	**.644**	**.791**	**.774**	**.788**	**.791**	**.788**	**.765**
	30th Percentile																									
SEA	.674	.605	.698	.698	.698	.698	.674	**.678**		.659	.634	.732	.732	.732	.732	.707	**.704**		.674	.605	.698	.698	.698	.698	.698	**.681**
OEA	.535	.535	.605	.605	.605	.651	.535	**.581**		.512	.439	.610	.561	.610	.585	.537	**.551**		.651	.419	.605	.605	.581	.605	.605	**.581**
UOE	.581	.512	.651	.651	.651	.581	.628	**.608**		.683	.463	.659	.634	.659	.659	.659	**.631**		.674	.512	.651	.651	.651	.535	.698	**.625**
ROE	.581	.558	.651	.651	.581	.651	.628	**.615**		.659	.537	.659	.634	.659	.488	.634	**.610**		.535	.488	.651	.651	.581	.651	.651	**.601**
WLEIS Total	.535	.512	.721	.721	.721	.721	.674	**.658**		.488	.561	.732	.732	.732	.732	.732	**.672**		.698	.698	.721	.721	.721	.721	.721	**.714**
Well-being	.395	.605	.721	.721	.721	.721	.698	**.654**		.585	.537	.732	.732	.732	.683	.732	**.676**		.628	.558	.721	.721	.721	.721	.721	**.684**
Self-control	.614	.477	.682	.682	.682	.682	.682	**.643**		.537	.512	.707	.707	.634	.634	.707	**.634**		.628	.488	.698	.698	.767	.698	.721	**.671**
Emotionality	.591	.545	.705	.705	.659	.705	.682	**.656**		.512	.512	.707	.634	.707	.707	.683	**.638**		.651	.488	.698	.628	.698	.698	.698	**.651**
Sociability	.545	.614	.682	.545	.682	.568	.545	**.597**		.561	.488	.683	.683	.683	.683	.659	**.634**		.558	.558	.674	.605	.698	.581	.628	**.615**
TEI Total	.488	.651	.721	.721	.721	.721	.721	**.678**		.659	.537	.732	.732	.732	.732	.732	**.693**		.674	.628	.721	.721	.721	.558	.744	**.681**
**Sub-Mean**	**.554**	**.561**	**.684**	**.670**	**.672**	**.670**	**.647**	**.637**		**.585**	**.522**	**.695**	**.678**	**.688**	**.663**	**.678**	**.644**		**.637**	**.544**	**.684**	**.670**	**.684**	**.647**	**.688**	**.650**
	40th Percentile																									
SEA	.477	.477	.568	.545	.545	.636	.614	**.552**		.512	.659	.610	.610	.585	.634	.585	**.599**		.535	.605	.581	.581	.535	.628	.465	**.561**
OEA	.605	.442	.535	.419	.628	.465	.535	**.518**		.561	.488	.585	.537	.659	.707	.537	**.582**		.698	.442	.512	.558	.674	.488	.651	**.575**
UOE	.372	.558	.581	.488	.442	.535	.512	**.498**		.537	.488	.561	.341	.463	.610	.341	**.477**		.442	.535	.581	.581	.442	.651	.535	**.538**
ROE	.442	.581	.581	.581	.628	.674	.535	**.575**		.463	.634	.610	.610	.512	.537	.561	**.561**		.512	.512	.512	.488	.558	.651	.512	**.535**
WLEIS Total	.488	.605	.605	.628	.605	.535	.535	**.571**		.463	.585	.610	.707	.488	.659	.537	**.578**		.628	.674	.605	.605	.605	.674	.651	**.635**
Well-being	.455	.591	.591	.591	.591	.455	.500	**.539**		.537	.463	.610	.561	.537	.585	.561	**.551**		.558	.465	.581	.581	.535	.581	.605	**.558**
Self-control	.488	.442	.581	.512	.465	.558	.488	**.505**		.512	.585	.585	.512	.634	.561	.537	**.561**		.488	.512	.558	.465	.395	.558	.488	**.495**
Emotionality	.568	.568	.568	.568	.636	.636	.523	**.581**		.610	.634	.610	.561	.610	.610	.585	**.603**		.465	.465	.512	.535	.512	.442	.581	**.502**
Sociability	.535	.558	.605	.605	.581	.488	.605	**.568**		.585	.512	.610	.610	.610	.585	.610	**.589**		.512	.512	.605	.605	.535	.651	.628	**.578**
TEI Total	.591	.568	.591	.614	.636	.500	.545	**.578**		.634	.537	.610	.634	.537	.585	.610	**.592**		.512	.535	.605	.581	.581	.581	.488	**.555**
**Sub-Mean**	**.502**	**.539**	**.581**	**.555**	**.576**	**.548**	**.539**	**.549**		**.541**	**.559**	**.600**	**.568**	**.563**	**.607**	**.546**	**.569**		**.535**	**.526**	**.565**	**.558**	**.537**	**.591**	**.560**	**.553**
	50th Percentile																									
SEA	.523	.614	.500	.455	.477	.477	.455	**.500**		.610	.537	.512	.463	.488	.537	.537	**.526**		.605	.535	.605	.535	.488	.488	.628	**.555**
OEA	.605	.442	.535	.419	.628	.465	.535	**.518**		.561	.488	.585	.537	.659	.707	.537	**.582**		.698	.442	.512	.558	.674	.488	.651	**.575**
UOE	.535	.535	.535	.535	.651	.558	.581	**.561**		.634	.415	.537	.585	.488	.610	.585	**.551**		.581	.605	.581	.558	.581	.605	.605	**.588**
ROE	.659	.591	.591	.591	.545	.591	.591	**.594**		.634	.512	.585	.537	.610	.585	.561	**.575**		.628	.535	.488	.465	.488	.512	.488	**.515**
WLEIS Total	.512	.628	.512	.744	.512	.535	.558	**.571**		.634	.561	.488	.537	.512	.537	.610	**.554**		.535	.535	.535	.512	.581	.512	.512	**.532**
Well-being	.568	.545	.477	.523	.523	.477	.500	**.516**		.488	.463	.561	.512	.512	.488	.512	**.505**		.535	.465	.488	.465	.442	.372	.395	**.452**
Self-control	.386	.318	.455	.432	.500	.568	.432	**.442**		.537	.439	.585	.683	.585	.634	.585	**.578**		.535	.558	.419	.465	.558	.442	.488	**.495**
Emotionality	.488	.558	.535	.535	.488	.488	.512	**.515**		.537	.610	.488	.561	.537	.512	.610	**.551**		.605	.558	.535	.442	.488	.558	.605	**.542**
Sociability	.523	.636	.568	.705	.432	.500	.568	**.562**		.634	.463	.561	.610	.585	.610	.585	**.578**		.535	.395	.581	.581	.512	.581	.558	**.535**
TEI Total	.512	.488	.512	.512	.581	.535	.488	**.518**		.634	.463	.488	.512	.634	.537	.512	**.540**		.465	.512	.442	.512	.442	.442	.419	**.462**
**Sub-Mean**	**.531**	**.536**	**.522**	**.545**	**.534**	**.520**	**.522**	**.530**		**.590**	**.495**	**.539**	**.554**	**.561**	**.576**	**.563**	**.554**		**.572**	**.514**	**.519**	**.509**	**.526**	**.500**	**.535**	**.525**
	60th Percentile																									
SEA	.488	.605	.651	.651	.651	.651	.605	**.615**		.512	.707	.634	.634	.634	.634	.585	**.620**		.558	.628	.651	.651	.605	.651	.581	**.618**
OEA	.455	.591	.659	.591	.636	.636	.523	**.584**		.610	.732	.610	.610	.634	.610	.634	**.634**		.581	.721	.628	.628	.651	.628	.605	**.635**
UOE	.636	.568	.614	.568	.500	.636	.545	**.581**		.561	.659	.634	.634	.610	.634	.537	**.610**		.581	.581	.605	.605	.581	.628	.651	**.605**
ROE	.659	.591	.591	.591	.545	.591	.591	**.594**		.634	.512	.585	.537	.610	.585	.561	**.575**		.628	.535	.488	.465	.488	.512	.488	**.515**
WLEIS Total	.512	.674	.651	.744	.674	.651	.651	**.651**		.585	.561	.634	.585	.634	.634	.537	**.596**		.581	.605	.651	.558	.651	.721	.605	**.625**
Well-being	.442	.419	.605	.488	.581	.605	.605	**.535**		.512	.561	.585	.585	.585	.585	.561	**.568**		.442	.488	.605	.535	.512	.535	.512	**.518**
Self-control	.477	.455	.591	.659	.568	.500	.568	**.545**		.561	.585	.585	.585	.610	.415	.610	**.564**		.558	.535	.581	.581	.535	.605	.605	**.571**
Emotionality	.500	.591	.591	.568	.545	.523	.545	**.552**		.585	.512	.585	.561	.439	.585	.488	**.537**		.581	.558	.605	.605	.488	.581	.512	**.561**
Sociability	.500	.409	.477	.500	.455	.568	.523	**.490**		.512	.585	.585	.585	.561	.585	.512	**.561**		.535	.395	.628	.628	.581	.698	.605	**.581**
TEI Total	.512	.442	.605	.535	.512	.465	.535	**.515**		.610	.439	.585	.585	.585	.561	.488	**.551**		.488	.581	.605	.605	.581	.512	.535	**.558**
**Sub-Mean**	**.518**	**.534**	**.603**	**.590**	**.567**	**.583**	**.569**	**.566**		**.568**	**.585**	**.602**	**.590**	**.590**	**.583**	**.551**	**.582**		**.553**	**.563**	**.605**	**.586**	**.567**	**.607**	**.570**	**.579**
	70th Percentile																									
SEA	.523	.750	.773	.773	.773	.727	.705	**.718**		.537	.707	.756	.756	.756	.756	.756	**.718**		.721	.674	.767	.767	.767	.767	.767	**.748**
OEA	.535	.512	.744	.698	.744	.744	.744	**.674**		.561	.537	.732	.732	.732	.732	.732	**.679**		.721	.628	.744	.744	.744	.744	.744	**.724**
UOE	.605	.628	.698	.628	.698	.698	.674	**.661**		.585	.610	.683	.683	.683	.634	.683	**.652**		.581	.535	.698	.651	.698	.674	.651	**.641**
ROE	.659	.659	.750	.750	.750	.750	.750	**.724**		.634	.585	.732	.732	.732	.732	.732	**.697**		.605	.512	.744	.651	.698	.628	.721	**.651**
WLEIS Total	.545	.682	.705	.705	.705	.705	.705	**.679**		.512	.561	.683	.585	.610	.659	.634	**.606**		.651	.744	.698	.698	.721	.698	.698	**.701**
Well-being	.628	.674	.721	.674	.651	.651	.721	**.674**		.659	.561	.707	.707	.707	.707	.707	**.679**		.628	.558	.721	.721	.721	.628	.721	**.671**
Self-control	.500	.455	.727	.727	.705	.705	.705	**.646**		.585	.415	.732	.732	.707	.488	.732	**.627**		.674	.558	.721	.744	.721	.721	.698	**.691**
Emotionality	.614	.705	.727	.727	.727	.727	.705	**.705**		.634	.610	.732	.732	.683	.659	.732	**.683**		.535	.605	.721	.721	.721	.558	.698	**.651**
Sociability	.535	.581	.721	.721	.721	.721	.698	**.671**		.561	.610	.707	.683	.683	.610	.659	**.645**		.767	.558	.744	.744	.744	.744	.744	**.721**
TEI Total	.535	.535	.698	.698	.698	.698	.744	**.658**		.561	.463	.683	.683	.683	.683	.610	**.624**		.651	.558	.698	.698	.721	.674	.674	**.668**
**Sub-Mean**	**.568**	**.618**	**.726**	**.710**	**.717**	**.713**	**.715**	**.681**		**.583**	**.566**	**.715**	**.702**	**.698**	**.666**	**.698**	**.661**		**.653**	**.593**	**.726**	**.714**	**.726**	**.684**	**.712**	**.687**
	80th Percentile																									
SEA	.614	.682	.841	.841	.841	.818	.841	**.782**		.732	.634	.829	.829	.829	.829	.829	**.787**		.814	.721	.837	.837	.837	.837	.837	**.817**
OEA	.814	.535	.837	.814	.837	.837	.837	**.787**		.659	.561	.829	.829	.805	.829	.829	**.763**		.860	.791	.837	.837	.837	.767	.837	**.824**
UOE	.818	.864	.909	.909	.909	.909	.909	**.890**		.756	.854	.902	.902	.902	.902	.902	**.875**		.907	.860	.907	.907	.907	.907	.907	**.900**
ROE	.674	.744	.837	.837	.837	.791	.837	**.794**		.683	.732	.829	.829	.829	.732	.829	**.780**		.744	.558	.814	.814	.814	.814	.814	**.767**
WLEIS Total	.628	.767	.814	.814	.814	.698	.767	**.757**		.756	.659	.805	.805	.805	.805	.805	**.777**		.791	.698	.814	.814	.814	.814	.814	**.794**
Well-being	.636	.591	.795	.795	.795	.614	.795	**.718**		.610	.585	.780	.780	.780	.780	.780	**.728**		.767	.721	.791	.791	.791	.791	.791	**.777**
Self-control	.605	.744	.837	.837	.837	.837	.837	**.791**		.756	.829	.829	.829	.829	.683	.829	**.798**		.721	.558	.837	.837	.837	.837	.837	**.781**
Emotionality	.605	.698	.814	.791	.814	.814	.767	**.757**		.707	.732	.805	.805	.805	.805	.805	**.780**		.721	.651	.814	.814	.814	.814	.814	**.777**
Sociability	.727	.750	.841	.818	.841	.705	.818	**.786**		.780	.756	.829	.829	.829	.829	.829	**.812**		.814	.791	.860	.837	.860	.860	.860	**.841**
TEI Total	.591	.636	.795	.705	.795	.795	.795	**.731**		.732	.683	.805	.805	.805	.805	.805	**.777**		.721	.674	.791	.791	.791	.767	.791	**.761**
**Sub-Mean**	**.671**	**.701**	**.832**	**.816**	**.832**	**.782**	**.821**	**.779**		**.717**	**.702**	**.824**	**.824**	**.822**	**.800**	**.824**	**.788**		**.786**	**.702**	**.830**	**.828**	**.830**	**.821**	**.830**	**.804**

*Note.* SEA = Self-emotion appraisal, OEA = Others’ emotion appraisal, UOE = Use of emotion, ROE = Regulation of emotion, WLEIS = Wong and Law emotional intelligence scale, TEI = Trait emotional intelligence. NB = Naïve Bayes models, SVM_*L*_ = Support Vector Machine linear models, SVM_*R*_ = Non-linear Support Vector Machine with a radial kernel function, SVM_*P*_ = Non-linear Support Vector Machine with a polynomial kernel function, KNN = k-Nearest Neighbors models, DT = Decision Tree models; RF = Random Forest models. Shaded areas highlight the accuracy results of the SVM_*R*_ models.

### 3.4. The Amount of Data Needed to Identify EI with ML Models

**Figure 6:**
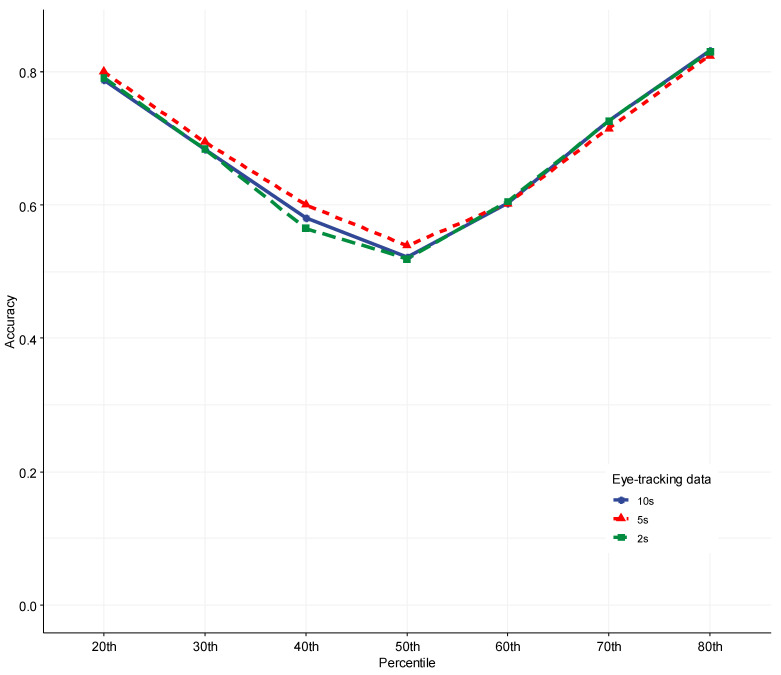
Comparative ML Performance Across Three Different Amounts of Eye-Tracking Data.

In order to determine if the amount of data impacted the ML accuracy, we replicated the analysis with three interest periods (i.e., three durations of eye-tracking data): 10 s, 5 s, and 2 s. We then aggregated the results across all the EI facets/measures using the SVM*_R_* model, the best-performing ML model discovered in the current study. Finally, we visualized and presented the results in Figure 6. 

Surprisingly, the results showed that interest periods seemed to have little impact on the ML model accuracy—all three interest periods yielded a highly similar accuracy. However, the 5 s interest period generated a slightly higher accuracy than the 10 s or 2 s interest period when the cutoff percentile was 50th or lower. When the cutoff percentile was 60th or higher, the 2 s and 10 s interest periods yielded almost identical accuracy. Nevertheless, the accuracy difference among the three interest periods was negligibly small. One-way ANOVA analysis also confirmed it: the accuracy among the three interest periods was not statistically significantly different, *F*(2, 207) = 0.295, *p* = .745, $\eta_{g}^{2}$ = 0.003.

### 3.5. The Most Predictive Eye-Tracking Features

**Figure 7:**
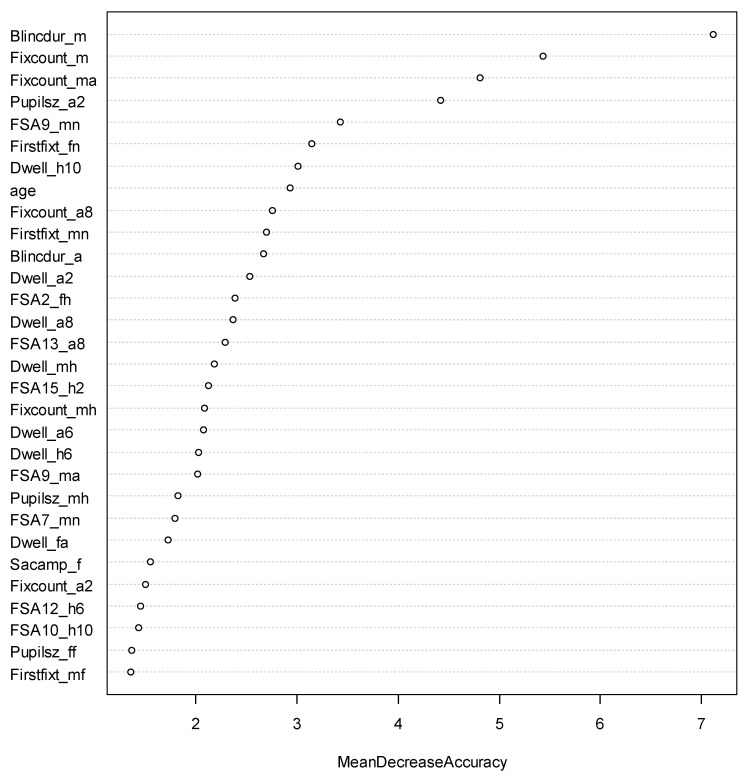
Variable Importance in Classifying Use of Emotion (UOE). Blincdur_m and Blincdur_a refer to average duration in milliseconds of all blinks in the trials of male and angry interest areas, respectively; Fixcount_m, Fixcount_ma, Fixcount_a8, Fixcount_mh, and Fixcount_a2 refer to the total number of fixations in male images, interest areas of angry male faces, angry faces in the 8:4 angry-to-happy face images, happy male faces, and angry faces in the 2:10 angry-to-happy face images, respectively; Firstfixt_fn, Firstfixt_mn, and Firstfixt_mf refer to the time when the first fixation started on the female neutral faces, male neutral faces, and male fearful faces, respectively; Pupilsz_a2, Pupilsz_mh, and Pupilsz_ff referr to the average pupil size across all fixations in the 2 angry faces in the trials of 2:10 angry-to-happy face images, male happy faces, and female fearful faces, respectively; FSA9_mn, FSA2_fh, FSA13_a8, FSA15_h2, FSA9_ma, FSA7_mn, FSA12_h6, and FSA10_h10 refer to the number of fixation sequences moving within the male neutral interest area in the trials of male images, from female angry interest area to female happy interest area, within the 8 angry faces in the trials of 8:4 angry-to-happy face images, within the 2 happy faces in the trials of 10:2 angry-to-happy face images, from male neutral interest area to male angry interest area, from male fearful interest area to neutral interest area, and within the 6 angry faces in the trials of 6:6 angry-to-happy face images, respectively. Dwell_h10, Dwell_a2, Dwell_a8, Dwell_mh, Dwell_a6, Dwell_h6, and Dwell_fa refer to the summation of the duration across all fixations on the 10 happy faces in the trials of 2:10 angry-to-happy face images, the 2 angry faces in the trials of 2:10 angry-to-happy face images, the 8 angry faces in the trials of 8:4 angry-to-happy face images, the male happy face interest areas, 6 angry faces in the trials of 6:6 angry-to-happy face images, and 6 happy faces in the trials of 6:6 angry-to-happy face images, respectively. Sacamp_f referred to the average size (in degrees of visual angle) of all saccades in the trial of female images.

**Figure 8:**
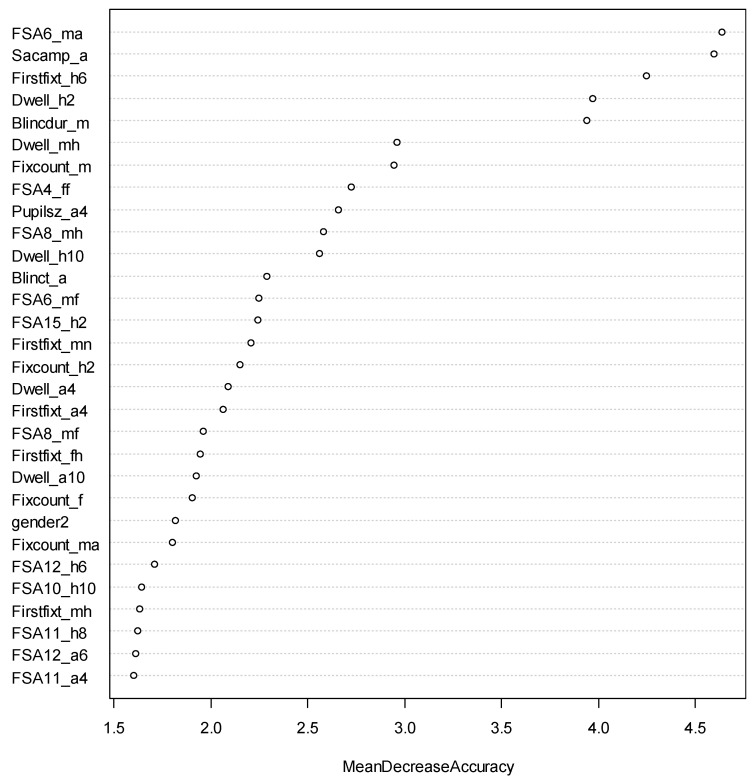
Variable Importance in Classifying Sociability. FSA6_ma, FSA4_ff, FSA8_mh, FSA6_mf, FSA15_h2, FSA8_mf, FSA12_h6, FSA10_h10, FSA11_h8, FSA12_a6, and FSA11_a4 refer to the number of fixation sequences moving within male angry faces, from female happy faces to female fearful faces, within male happy faces, from male angry to male fearful faces, within happy faces in the 10:2 angry to happy face trials, from male happy to male fearful faces, from angry faces to happy faces in the trail 6:6 angry to happy face trials, from angry faces to happy faces in the 2:10 angry to happy face trials, from angry to happy faces in the 4:8 angry to happy face trials, from angry to happy faces in the 6:6 angry to happy face trials, and within angry faces in the 4:8 angry to happy face trials, respectively; Sacamp_a refers to the average amplitude (in degrees of visual angle) of all saccades in the trials of crowded face images; Firstfixt_h6, Firstfixt_mn, Firstfix_ a4, Firstfixt_fh, and Firstfixt_mh refer to the starting time of the first fixation to enter the happy face interest area in the trials of 6:6 angry-to-happy face trials, the male neutral faces, the angry faces in the trials of 4:8 angry-to-happy face trials, and the female happy faces, respectively; Dwell_h2, Dwell_mh, Dwell_h10, Dwell_a4, and Dwell_a10 refer to the summation of the duration across of fixations on the happy face interest area in the 10:2 angry-to-happy face trials, the male happy faces, the happy face interest area in the 2:10 angry-to-happy face trials, the angry face interest area in the 4:8 angry-to-happy face trials, and the angry face interest area in the 10:2 angry-to-happy face trials, respectively; Blincdur_m and Blinct_a refer to the average duration in milliseconds of all blinks in the trials of male images and angry interest areas, respectively; Fixcount_m, Fixcount_h2, Fixcount_f, and Fixcount_ma refer to the total number of fixations on the male images, happy faces in the 10:2 angry-to-happy face trials, female faces, and male angry faces, respectively. Pupilsz_a4 refers to the average pupil size across all fixations in the four angry faces in the trials of 4:8 angry-to-happy face images.

Lastly, we further investigated which eye-tracking measures were most predictive of EI by conducting variable importance analyses. We conducted such analyses for two EI facets: Use of Emotion (UOE) from the WLEIS measure and Sociability from the TEIQue-SF measure, as those two facets showed relatively high accuracy in the current study. The variable importance was measured by Mean Decrease Accuracy, the averaged decrease in model accuracy in predicting the EI outcome when a specific variable was excluded from the model. We presented the top predictors in Figure 7 and Figure 8 for UOE and Sociability, respectively.

For the Use of Emotion (UOE; Figure 7) facet, the most predictive eye-tracking measures included the average duration (in milliseconds) of blinks in the trials of male images, the total number of fixations in the trials of male images, the total number of fixations in the anger interest area in the trials of male images, the average pupil size across all fixations viewing the two angry-face interest areas in the trials of 2:10 angry-to-happy face images, and the number of fixation movements within the male neutral interest area in the trials of male images. These predictors were all negatively associated with high EI scores, so lower eye measures (e.g., fewer fixations, smaller pupil size, etc.) are associated with higher EI scores.

**Figure 9:**
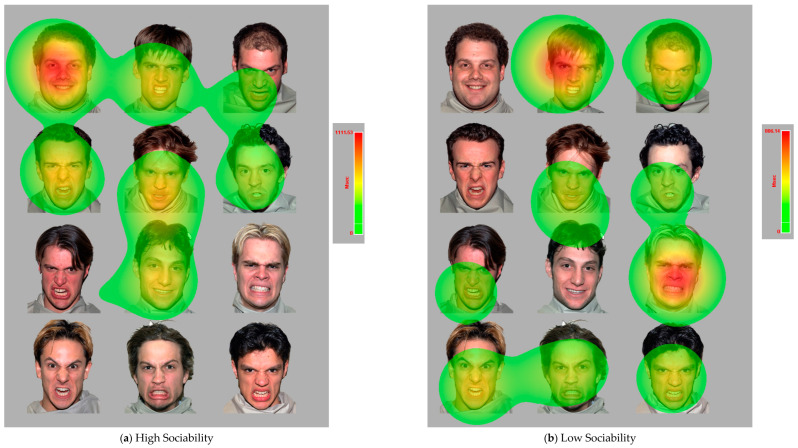
The Differed Amount of Time Fixating on the Happy Faces in 10:2 Angry-to-Happy Face Images Among Participants with a High (**a**) and Low (**b**) Sociability Score.

The most predictive eye-tracking measures for the Sociability facet are presented in Figure 8. It was found that the number of fixations continuing to focus on the male anger interest area in the trials of male images, the starting time of the first fixation to enter the happy face interest area in the trials of 6:6 angry to happy face images, and the average duration (in milliseconds) of all blinks in the trials of male images negatively predicted high Sociability scores, while the average amplitude (in degrees of visual angle) of all saccades in the trials of crowded face images and the summation of the duration across all fixations on the happy face interest area in the trials of 10:2 angry-to-happy face images positively predicted high Sociability scores. This difference in the fixation duration on the happy face interest areas in the 10:2 angry-to-happy images is further illustrated in Figure 9, where participants with a higher Sociability score viewed the happy faces for a longer time than the time they viewed the angry faces (Figure 9a), while participants with a lower Sociability score viewed the angry faces for a longer duration than the time they viewed the happy faces.

## 4. Discussion

With the accelerating development and application of AI technology, many industries have witnessed exciting revolutions in recent years, and this revolution is also happening in the psychometric discipline. With the increasing application of AI techniques and methodologies to psychometrics, a new research field—Psychometric AI—is burgeoning (Bringsjord 2011; Bringsjord and Schimanski 2003). Early research in this area has demonstrated the clear advantages of this new field: AI can help improve measurement accuracy, efficiency, and effectiveness (Chen et al. 2010; Hussein et al. 2019; Mujtaba and Mahapatra 2020) and also reduce human bias and increase objectivity in the measurement (Altemeyer 2019; Wang et al. 2022). For personality measurement specifically, existing research has revealed that ML models could judge personality traits more accurately than human beings (Wu et al. 2015), and AI-driven classifiers could predict self-reported personality scores with an accuracy of up to 90% (Berkovsky et al. 2019).

The current study contributes to the literature by applying Psychometric AI to measure emotional intelligence. To our knowledge, this is the first study in the Psychometric AI field that systematically examined the accuracy of various machine learning models. More importantly, leveraging eye-tracking techniques, the current research explored a non-invasive sensing technology capable of identifying emotional intelligence at the individual level with decent predictive accuracy.

The results from 1470 ML runs revealed that the performance of ML models depended on the percentiles to dichotomize emotional intelligence scores. With a dichotomy at the 20th or 80th percentile, the accuracy could be around or above 80%. However, when the data were dichotomized around a middle point (e.g., at the 40th–60th percentile), the accuracy was modest, between 50 and 60%. This finding indicated that the ML models were better at identifying emotional intelligence scores at the bottom or the top. This is an interesting discovery because, on the one hand, it makes sense that both lower and higher scores are relatively easily predicted as they are more extreme; however, on the other hand, such a dichotomy creates imbalanced classification, which is typically more challenging for ML models (Brownlee 2020). Nevertheless, machine learning models achieved an average accuracy ranging from 54% to 82% in measuring binary scores of emotional intelligence in the current study. Although it was slightly lower than what was found by Berkovsky et al. (2019), such a performance is quite common in social sciences. For example, a recent study by Rasmussen et al. (2023) also produced a modest accuracy of 61% when they applied deep learning techniques to predict a binary outcome of political ideology (rightist vs. leftist) with a large sample of thousands of individuals. 

Perhaps one of the most encouraging findings from this study was the discovery that AI models could achieve decent accuracy with as little as 2 or 5 s of eye-tracking data. This finding is strikingly different from the principle of classic test theory (Allen and Yen 2001), which assumes that more data increase better measurement quality (i.e., higher measurement reliability and validity) due to the corresponding reduced measurement errors. Yet, our study seemed to imply that AI algorithms might be powerful enough to overcome measurement problems associated with data shortage, which is encouraging for the psychometric discipline as it saves time and monetary resources that would otherwise be required for data collection in the traditional approach. 

Interestingly, our study has revealed mixed results on the effects of emotional intelligence facets and measures on classification accuracy. There seemed to be unclear patterns regarding which emotional intelligence facets or measures could generate a higher or lower classification accuracy, suggesting that they had a much smaller effect on the accuracy compared to dichotomy percentiles and machine learning models. This finding might be due to the fact that all the facets and measures were highly affective, as Berkovsky et al. (2019) found that traits associated with affect were more predictable (with higher accuracy) than other traits (e.g., behavior- and cognition-based traits). Indeed, the less affective facets, such as Well-being and Self-control in the TEIQue-SF measure, produced slightly lower accuracy, as shown in Figure 5, consistent with Berkovsky et al. (2019). 

Lastly, our study found that many eye-tracking measures were highly predictive of EI scores, including the average duration of blinks, the total number of fixations in certain trials, the average pupil size, the starting time of the first fixation to enter the happy faces in crowd images, the total time fixating on the happy faces in crowd images which had overwhelmingly more angry faces than happy faces, etc. These findings are indeed consistent with the attentional bias discovered in the literature. That is, individuals with higher (vs. lower) EI scores tended to prefer (vs. avoid) positive visual stimuli such as happy faces (Al-Samarraie et al. 2022; Davis 2018; Lea et al. 2018). These consistent findings suggest that eye-tracking techniques are indeed ideal for emotional intelligence research.

As eye-tracking and AI techniques have become increasingly accessible in both industries and academia, we believe the findings from this study have important practical implications. Indeed, eye tracking has become popular in psychological and managerial research and made significant contributions to both theoretical advances and practical applications (e.g., Eckstein et al. 2017; Shishido et al. 2019; Valtakari et al. 2021; Madera and Hebl 2012). The current research implies that integrating AI techniques may further expand our understanding in this research area. For example, although researchers have studied faking behavior on personality inventories from various perspectives (e.g., Cao and Drasgow 2019; Scherbaum et al. 2013), it seems to be challenging to fundamentally eliminate this problem as it is inherent to self-report methods. The current study may shed light on this issue by integrating the unobtrusive eye-tracking sensing technology and Psychometric AI methodology.

Despite many notable novel contributions, the current study also came with several limitations. For example, the measures of emotional intelligence were only focused on the Trait EI construct. Future research may explore the measurement of Ability EI (e.g., abilities to understand and manage EI as measured by MacCann and Roberts 2008) through the eye-tracking and Psychometric AI perspectives. In addition, the ML models only analyzed the binary classification of EI measures in the current study. The multiclass classification ML models should be considered in future research. Similarly, deep learning, such as neural networks (Huang and Khan 2021), may be applied in the future to improve accuracy. In addition, the current analyses relied on the eye-tracking metrics derived from the gaze data. Future studies may analyze the sequential gaze data directly by using Long Short-Term Memory (LSTM) and the Transformer Architecture. In terms of visual stimuli, the current study only used Caucasian male faces in the face crowds, and faces of other races should be used in future studies, especially for participants with racially diverse backgrounds due to the race effect on visual attention (Kawakami et al. 2022). Lastly, future research may also add relevant videos (see Berkovsky et al. (2019) for an example) to combine with the image visual stimuli to improve both predictive accuracy and ecological validity.

## 5. Conclusions

The current study has explored a burgeoning field—Psychometric AI—that integrates artificial intelligence techniques and methodologies with psychometric assessments and testing, particularly its application to emotional intelligence measurement. Through performing 1470 ML runs, this study systematically examined the measurement accuracy of various machine learning models in predicting different facets/measures of emotional intelligence. The results also revealed that AI algorithms were powerful enough to achieve high accuracy with as little as 2 or 5 s of eye-tracking data. In addition, the current study has identified eye-tracking features that are effectively predictive of emotional intelligence scores, and many of the features are consistent with the literature. We believe these findings advance our current understanding of Psychometric AI for EI measurement and also shed important light on the practical implications for the applications of emotional intelligence in management and education.

## Figures and Tables

**Table 1:** Means, standard deviations, and correlations with confidence intervals.

Variable	M	SD	1	2	3	4	5	6	7	8	9	
Wong and Law Emotional Intelligence Scale (WLEIS): Facets and Measure												
1. SEA	5.39	1.00	.72									
2. OEA	5.49	0.93	.57 **	.66								
3. UOE	5.36	1.16	.49 **	.46 **	.80							
4. ROE	5.04	1.19	.54 **	.41 **	.65 **	.75						
5. WLEIS Total	5.32	0.86	.80 **	.73 **	.83 **	.83 **	.88					
Trait Emotional Intelligence Questionnaire (TEIQue-SF): Facets and Measure												
6. Well-being	5.33	1.07	.44 **	.30 **	.66 **	.46 **	.59 **	.81				
7. Self-control	4.49	1.04	.48 **	.22 **	.50 **	.65 **	.59 **	.51 **	.70			
8. Emotionality	5.01	0.89	.48 **	.39 **	.36 **	.45 **	.52 **	.46 **	.46 **	.61		
9. Sociability	4.58	1.03	.32 **	.26 **	.44 **	.33 **	.43 **	.53 **	.35 **	.43 **	.70	
10. TEIQue-SF Total	4.89	0.78	.56 **	.39 **	.67 **	.61 **	.70 **	.81 **	.74 **	.76 **	.73 **	.89

Note. SEA = Self-emotion appraisal, OEA = Others’ emotion appraisal, UOE = Use of emotion, ROE = Regulation of emotion. ** indicates *p* < .01.

## Data Availability

The data presented in this study are available from the corresponding author upon request.
